# Transcatheter edge-to-edge repair for tricuspid valve regurgitation in apolipoprotein A-I–associated cardiac amyloidosis: case report

**DOI:** 10.1093/ehjcr/ytad582

**Published:** 2023-11-30

**Authors:** Mohammad Alomari, Prajwal Reddy, Abdallah El Sabbagh, Peter Pollak, Melissa Lyle

**Affiliations:** Department of Cardiothoracic Surgery, Mayo Clinic, Jacksonville, FL, USA; Department of Cardiovascular Disease, Mayo Clinic, Jacksonville, FL, USA; Department of Cardiovascular Disease, Mayo Clinic, Jacksonville, FL, USA; Department of Cardiovascular Disease, Mayo Clinic, Jacksonville, FL, USA; Division of Advanced Heart Failure and Transplant Cardiology, Department of Transplantation, Mayo Clinic, 4500 San Pablo Road, Jacksonville, FL 32224, USA

**Keywords:** Amyloidosis, Tricuspid regurgitation, Right heart failure, MitraClip, Case report

## Abstract

**Background:**

Amyloidosis is defined by abnormal protein folding and subsequent deposition in tissues. Cardiac involvement is usually related to misfolded monoclonal immunoglobulin light chains or misfolded transthyretin; however, apolipoprotein A-1–associated amyloidosis is a hereditary form of amyloidosis resulting from mutations in the AAPOA1 gene that can also result in cardiac amyloidosis. Although there have been advancements in noninvasive algorithms for the diagnosis of cardiac amyloidosis, endomyocardial biopsy (EMB) may still be warranted. All individuals undergoing EMB are susceptible to complications, including tricuspid valve injury resulting in severe tricuspid valve regurgitation.

**Case summary:**

Our patient is a 70-year-old white man presented with symptoms of dyspnoea on exertion and decreased functional capacity, diagnosed previously with apolipoprotein A-I cardiac amyloidosis, confirmed by EMB. He developed progressive right-sided heart failure secondary to iatrogenic flail tricuspid leaflet related to the diagnostic EMB. He underwent a successful transcatheter tricuspid valve edge-to-edge repair with 4D intracardiac echocardiographic guidance. At the recent follow-up, the patient showed improved symptoms, with increased stamina, and transoesophageal echocardiography revealed a 65% ejection fraction and mild tricuspid regurgitation (TR).

**Discussion:**

Tricuspid valve injury is one of the complications associated with EMB, which can result in severe TR. Transcatheter tricuspid valve edge-to-edge repair can be a useful option for patients considered too high risk for surgical intervention, such as those with advanced cardiac amyloidosis.

**Learning points**

- To identify apolipoprotein A-I–related amyloidosis as a rare hereditary cause of cardiac amyloidosis.
- To outline the potential indications for transcatheter tricuspid valve edge-to-edge repair in patients with cardiac amyloidosis with severe symptomatic tricuspid valve regurgitation.
- To recognize the multidisciplinary approach in patients with cardiac amyloidosis and valvular heart disease, highlighting the use of transcatheter procedures and advanced imaging technology such as intracardiac echocardiography for interventional guidance.

## Introduction

Systemic amyloidosis is characterized by the accumulation of misfolded precursor proteins into insoluble amyloid fibrils with subsequent extracellular deposition into organs leading to dysfunction.^1^ Cardiac involvement can result in a restrictive cardiomyopathy, as well as atrial and ventricular tachyarrhythmias and bradyarrhythmias.^2^ Although over 35 different proteins are capable of aggregating into amyloid fibrils, only nine are known to infiltrate the heart and result in clinically significant cardiac amyloidosis.^2^ Immunoglobulin light chain (AL) and transthyretin (ATTR) amyloidosis remain the most common cause of cardiac amyloidosis.^2^ Apolipoprotein A-I (AApoAI)–associated amyloidosis is a rare hereditary form that can also affect the heart and has been cited as the third most common type of hereditary amyloidosis in the UK.^2^ Eighteen different AApoAI variants have been identified with evidence of renal, hepatic, and cardiac involvement, with renal and hepatic involvement being most common. The exact prevalence of AApoAI amyloidosis is unknown, but larger case series have been provided further information regarding the natural history and long-term outcomes of patients with hereditary AApoAI amyloidosis.^3^ Treatment is supportive given there are no disease-modifying therapies available for AApoAI amyloidosis.^4^

The diagnosis of AApoAI–associated amyloidosis can be challenging. There have been significant advancements in the noninvasive diagnosis of amyloidosis, particularly with bone scintigraphy being utilized to noninvasively diagnose ATTR amyloidosis if Grade 2 or 3 uptake is seen in the absence of a monoclonal protein.^2^ However, technetium-99m pyrophosphate (PYP) or 3,3-diphosphono-1,2-propanodicarboxylic acid (Tc-DPD) scanning may be negative in AApoAI-associated amyloidosis. If haematologic testing and scintigraphy are both negative, but clinical suspicion for amyloidosis persists, then it is important to consider cardiac magnetic resonance (CMR) in addition to endomyocardial biopsy (EMB) to confirm the diagnosis.^2^

Although EMB remains an important diagnostic tool if the diagnosis of cardiac amyloidosis cannot be made noninvasively, potential complications can arise, including cardiac perforation with resultant tamponade, electrical conduction disturbances including heart block, and tricuspid valve (TV) injury.^5^ The TV can be damaged at the valvular and subvalvular level during EMB, and the rate of valvular trauma during biopsy has been estimated at 0.02–1.1%.^6^ We report a case of iatrogenic flail tricuspid leaflet, secondary to TV injury during EMB, in a patient with AApoAI amyloidosis, who developed worsening right heart failure symptoms related to the severe tricuspid regurgitation (TR). He underwent a transcatheter TV edge-to-edge repair with 4D intracardiac echocardiography (ICE) guidance. Transcatheter TV repair in patients with ATTR cardiac amyloidosis has been described previously,^7^ but to our knowledge, this is the first reported case of TV transcatheter edge-to-edge repair (TEER) for a flail tricuspid leaflet in a patient with AApoAI amyloidosis utilizing 4D ICE to guide the transcatheter procedure.

## Summary figure

**Table:** 

Date	Event
**5 April 2018**	Patient referred to our institution for possible cardiac amyloidosis based on outside ECHO and magnetic resonance imaging (MRI) findings
**5 April 2018**	No monoclonal protein detected from haematologic testing, and nuclear scintigraphy was negative for transthyretin (TTR) amyloidosis
**26 April 2018**	Fat aspirate was positive for amyloid by Congo Red stain, but mass spectrometry (MS) unable to be completed on sample
**1 June 2018**	Admitted. Endomyocardial biopsy performed and complicated by flail TV leaflet. Pathology demonstrated amyloid deposits with liquid chromatography–MS (LC–MS) for amyloid typing revealing AApoAI amyloid with the Leu99Pro mutation. Genetic testing confirmed genetic variant
**26 March 2019**	On follow-up, the patient had an increase in bilateral lower limb oedema, and he was initiated on 20 mg furosemide daily
**16 November 2021**	Presented with shortness of breath. Computed tomography scan demonstrated bilateral small pleural effusions and ascites, requiring frequent paracenteses locally. Transoesophageal echocardiography (TEE) revealed ejection fraction (EF) 50% and severe TV regurgitation. The patient was admitted
**4 May 2022**	Transoesophageal echocardiography revealed EF of 55% and severe TR regurgitation
**29 June 2022**	Given progressive right heart failure in the setting of flail TV leaflet and restrictive cardiomyopathy from amyloidosis, discussed the role of TV edge-to-edge repair with interventional cardiology to improve symptoms
**30 August 2022**	Underwent successful transcatheter TV edge-to-edge repair
**15 September 2022**	Mild residual TV regurgitation, with significant improvement in symptoms and decreased interval need for paracenteses
**17 August 2023**	Transoesophageal echocardiography, EF of 65%, and mild residual TV regurgitation

## Case presentation

A 70-year-old white man with a history of coronary artery disease, paroxysmal atrial fibrillation, and sinus node dysfunction with prior permanent pacemaker implantation presented with symptoms of dyspnoea on exertion and decreased functional capacity.

The cardiovascular examination findings include a jugular venous pressure (JVP) that is elevated by 2 cm above the clavicle and the presence of a 3/6 systolic murmur on the left lower sternal border. The lungs were clear on auscultation. Transthoracic echocardiography revealed a left ventricular EF of 52%, increased biventricular wall thickness, bi-atrial enlargement, thickened atrioventricular valve leaflets with moderate mitral and mild TR, and abnormal global longitudinal strain of −9% with apical sparing. Cardiac MRI demonstrated extensive subendocardial delayed gadolinium. Laboratory evaluation revealed normal renal function (creatinine, 1.2 mg/dL, normal: 0.7–1.3 mg/dL), high-sensitivity troponin 61 ng/mL (normal: <14 ng/mL), NT-proBNP 1965 µg/mL (normal: <125 µg/mL), total bilirubin 2.7 mg/dL (normal: <1 mg/dL), direct bilirubin 0.7 mg/dL (normal: 0–0.3 mg/dL), and a normal haematologic evaluation with normal free AL ratio and no monoclonal protein detected on serum or urine immunofixation. Nuclear scintigraphy utilizing technetium-labelled PYP (Tc-99m-PYP) was performed and negative for ATTR amyloidosis. Fat pad aspirate did reveal amyloid deposits by Congo Red staining, but there was insufficient number of amyloid-associated peptides to perform amyloid typing. Given high clinical suspicion of cardiac amyloidosis, EMB was recommended,^7^ and biopsy revealed amyloid deposits by Congo Red staining with LC–MS demonstrating AApoAI amyloidosis, and genetic testing confirmed the Leu99Pro mutation.

During the EMB, there was injury to the TV consisting of a flail septal leaflet with severe eccentric TR (see Supplementary material online, *Videos S1* and *S2*). The patient was initially managed conservatively with diuretics. However, over 3 years, he developed worsening dyspnoea, decrease in functional capacity, autonomic dysfunction felt to be related to the AApoAI amyloidosis, and recurrent ascites requiring paracenteses, early satiety, and worsening symptoms of right heart failure with New York Heart Association (NYHA) IIIb symptoms. Although some of his symptoms were related to progression of his restrictive cardiomyopathy secondary to cardiac amyloidosis, his severe TR was felt to be contributing to the rapid progression of right heart failure symptoms. Repeat transthoracic echocardiography revealed moderate–severe right atrial enlargement, increased right ventricular wall thickness, right ventricular end-diastolic diameter at the base was 4.8 cm, TV annulus systolic excursion by M-Mode (TAPSE) 13 mm, and severe TV regurgitation with hepatic vein systolic flow reversals and a dilated and noncollapsible inferior vena cava. Given the presence of advanced cardiac amyloidosis, after multidisciplinary discussion with cardiovascular surgery, structural cardiology, and shared decision-making with the patient, the decision was made to proceed with TV TEER to alleviate some of his right-sided heart failure symptoms.

Patient successfully underwent TV TEER with off-label use of the MitraClip device (Abbott Vascular, Santa Clara, CA, USA). The MitraClip device was chosen given lack of availability of the PASCAL and the TriClip. The MitraClip delivery system was inserted via a right femoral vein approach. Given suboptimal TEE images, 4D ICE catheter was introduced via a left femoral vein with catheter tip positioned in the right atrium. With ICE guidance, the MitraClip XTW clip was implanted with a successful grasp of the septal and anterior leaflets (see Supplementary material online, *Videos S3* and *S4*). The degree of TR was reduced to mild with closure of the clip and remained stable after the release. The patient was administered post-procedural anticoagulation therapy in the form of apixaban. Post-procedural transthoracic echocardiogram 1 day post-procedure demonstrated mild TR with a TV diastolic mean Doppler gradient 2 mmHg (heart rate of 80 b.p.m.). Patient did require intravenous diuretics prior to hospital discharge but remains on a stable diuretic regimen in the outpatient setting with improved functional capacity, NYHA II. On the most recent follow-up visit, the patient reported an improvement in their symptoms, including increased stamina. Transoesophageal echocardiography revealed EF of 65% and mild TR.

## Discussion

Although noninvasive diagnosis of cardiac amyloidosis is possible, this typically refers to the diagnosis of ATTR amyloidosis with bone scintigraphy in the absence of a monoclonal protein.^2^ Apolipoprotein A-I–associated amyloidosis is a rare hereditary form of amyloidosis that can have cardiac involvement.^2^ Given diagnostic challenges, an EMB may be warranted to confirm the diagnosis of AApoAI-associated cardiac amyloidosis. The complications of EMB have been well described and include right ventricular perforation and pericardial tamponade, electrical conduction disturbances, and TV injury.^8^ When the TV injury results in severe TV regurgitation, intervention may be warranted.

Multiple studies have demonstrated the benefit of an aggressive surgical approach to the correction of severe TV regurgitation in general, but there is an associated high in-hospital death rate, explaining why only a small percentage of patients undergo surgical treatment.^9^ Prior reports have highlighted the role for transcatheter TV repair to address symptomatic severe TR in patients who are deemed high surgical risk.^10^ An observational study by Nickenig *et al.*^11^ highlighted the benefit of transcatheter TV repair for symptomatic severe TR in high surgical risk patients. More recently, Sorajja *et al.*^12^ demonstrated the TV TEER utilizing the TriClip Transcatheter Tricuspid Valve Repair system (Abbott Structural Heart) to be safe and effective at reducing severity of TV regurgitation and improving functional capacity in the TRILUMINATE Pivotal randomized controlled trial. As described in our case, ICE can be utilized for additional imaging guidance during TV TEER when TEE image quality is insufficient, and the role of ICE in such situations has been demonstrated in prior studies.^13^

Less is known about the role of TEER to address severe TV regurgitation in patients with cardiac amyloidosis. Because TV regurgitation is usually secondary to right ventricular remodelling in the setting of restrictive cardiomyopathy, the utility of TV intervention in cardiac amyloidosis is questionable.^14^ Hoerbrand *et al.*^7^ described their experience with eight wild-type ATTR cardiac amyloid patients undergoing transcatheter TV repair for severe TV regurgitation, and device success rates in the amyloidosis group (75%) were comparable to those in the control group (86%) with a *P* = 0.597. The overall probability of hospitalization or death in the amyloidosis group (13%) was also similar to that in the control group (25%, *P* = 0.646) at the 3-month follow-up. Transcatheter TV repair resulted in improved NYHA functional class (*P* = 0.031), and a 6-min walking distance increase from 313 ± 118 to 337 ± 106 (*P* = 0.012). However, further studies are needed to elucidate the role of TV transcatheter repair, including which transcatheter technique is preferred and what mechanism of TV injury is most amenable to intervention, in patients with cardiac amyloidosis and severe TV regurgitation. To our knowledge, our case is the first reported case of TV TEER in a patient with AApoAI amyloidosis associated with the Leu99Pro mutation, also highlighting the use of 4D ICE technology to provide high-quality images to guide the procedure.

## Conclusion

This is the first reported case of successful transcatheter TV edge-to-edge repair of an iatrogenic flail tricuspid leaflet with 4D ICE guidance in a patient with AApoAI-related cardiac amyloidosis. Transcatheter TV repair in amyloid patients is an area of interest that requires further research because these individuals are at high risk for open surgery. However, if performed in specialized centres and by qualified physicians, it may be a safe and effective method for symptomatic relief.

## Supplementary Material

ytad582_Supplementary_Data

## Data Availability

The data underlying this manuscript are available within the article and in its online Supplementary material.

## Lead author biography

Dr Mohammad Alomari is a research fellow at Mayo Clinic in Jacksonville, FL, who aspires to pursue a residency in cardiothoracic surgery.

## Supplementary material

**Consent:** The authors confirm that written informed consent for the publication of this case report was obtained from the patient in line with the Committee on Publication Ethics (COPE) guidelines.

**Funding:** None declared.
